# Periodontal ageing and its management via pharmacological glucose modulation

**DOI:** 10.3389/fdmed.2024.1415960

**Published:** 2024-07-01

**Authors:** Vitor C. M. Neves, Viktor Savchenko, James Daly, Paul Sharpe

**Affiliations:** ^1^ Restorative Dentistry Unit, the School of Clinical Dentistry, University of Sheffield, Sheffield, United Kingdom; ^2^ Healthy Lifespan Institute, University of Sheffield, Sheffield, United Kingdom; ^3^ Oxford Uehiro Centre for Practical Ethics, University of Oxford, Oxford, United Kingdom; ^4^ Department of Civil Law Disciplines, V.N. Karazin Kharkiv National University, Kharkiv, Ukraine; ^5^ Bristol Dental Hospital, University Hospitals Bristol and Weston NHS Foundation Trust, Bristol, United Kingdom; ^6^ Centre for Craniofacial and Regenerative Biology, FoDOCS, King’s College London, London, United Kingdom; ^7^ Institute of Animal Physiology and Genetics, Brno, Czech Republic

**Keywords:** periodontal disease, metformin, ageing, preventive medicine, glycaemic control, multimorbidity prevention

## Abstract

Periodontal disease (PD), a widespread non-communicable disease, affects over 90% of the global population with no known cure. Current management strategies focus on the stabilisation of disease progression, which is successfully achieved to a limited extent. Yet the never-ending battle between bacteria and the gingiva involves a complex interplay between genetic, microbial and environmental factors, demanding innovative approaches to improve the prevention and stabilisation of this disease. Glucose is the body's source of energy and research has shown that dysregulation of the glucose metabolism impacts PD establishment and progression, as well as the development of systemic non-communicable diseases. Metformin, a drug known for its efficacy in diabetes treatment via controlling glucose metabolism, also demonstrated cardioprotective effects, increased longevity, and anti-inflammatory properties. Metformin has been used in gel format in clinical trials for non-surgical treatment of PD, however, its systemic use in normoglycemic individuals with PD is less explored. A recent study presented compelling evidence of metformin's preventive potential, impacting PD and markers of systemic health involved in metabolic health linked to improvement of lifespan. Therefore, this review discusses the aspects of ageing as a concept in the periodontium and the potential benefits of modulating glucose metabolism through metformin to prevent PD, indirectly preventing systemic conditions involved in multi-morbidity, addressing a critical gap in current management. It also examines the choice between implementation of behaviour change and/or medication as a strategy to add to current oral hygiene strategies. Finally, it discusses the ethical implications of prescribing systemic medication in dentistry.

## Graphical Abstract

The never-ending battle between gingiva and biofilm. Characters of the illustration: Yellow fighter, Bac; Red fighter, Gingiva; Toothbrush, Coach Toothy; Bone with cap, Coach Bony; Round Tablet with Lightening sign, Coach metformin; Orange circle in the audience, Glucose; Purple triangle in the audience, Insulin; Yellow round crusty characters in the audience, Bacteria.

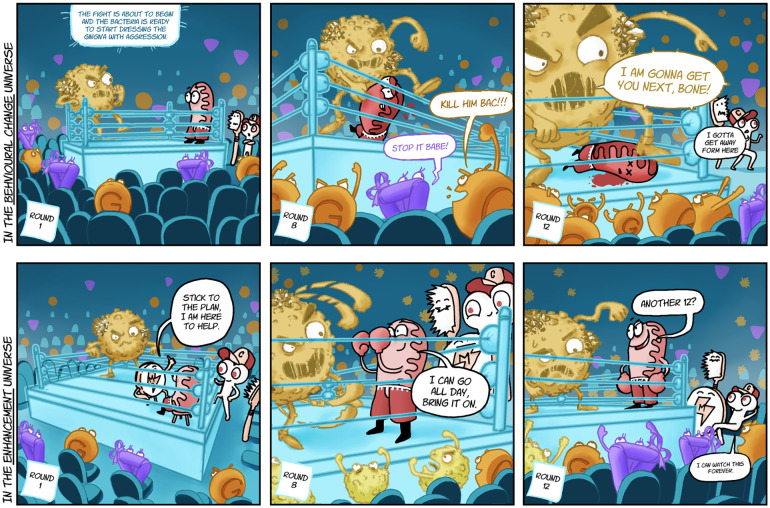

## Introduction

Periodontal disease (PD), in any form, affects over 90% of the global population (1). Unfortunately, PD has no cure known to date, and the only treatment available is to stabilize it to avoid progression. Given that PD develops as a result of gene-environmental interaction, a cure will only be available when capacity-altering, biologically-based interventions are safe and compatible with the demands of distributive justice (2, 3). In light of PD's epidemiology and complex pathogenesis, seeking to implement curative strategies may not be currently the best way to tackle the global burden of the disease. Therefore, it becomes clear that there is a need to enhance prevention and stabilization of PD via the development of novel and accessible strategies to complement current methodologies.

PD is a non-communicable disease (NCD) with direct links to systemic health, including some life-threatening diseases, such as cardiovascular diseases, diabetes, and obesity (4). Commonly, these conditions develop during one's lifespan, and the risk of these conditions tends to increase with age, influenced by a combination of genetic, environmental, and lifestyle factors (5). A significant proportion of the population develops PD at a younger age (30 years old) when compared to other systemic NCDs (6, 7). Given the similarities between the host's metabolic-mediated inflammatory response driving PD progression and other NCDs, early systemic management of PD may reduce the impact of these systemic diseases on individuals, improving public health.

With the increasing life expectancy and a growing proportion of elderly individuals in the general population, there is a pressing need to comprehend the reasons behind the escalating susceptibility to chronic morbidity, disability, and frailty associated with ageing, making it a key public health concern. Given the high prevalence of PD and its association with ageing, further understanding on how ageing affects the oral tissues and microbiome is essential to evolving dentistry beyond the current standard of practice, where management of disease does not account for ageing.

Most recently, glucose metabolism modulation has been indicated as a key enhancer of longevity by decreasing NCD incidence (8–10), and in PD literature, it is widely known that dysregulation of glucose metabolism accelerates PD progression. Biofilm build-up and glycaemic peaks are both transient physiological factors of human physiology, and current PD management strategies do not tackle glycemia nor have an impact on overall systemic NCD prevention. Therefore, this article investigates the potential of implementation of glucose metabolism modulation in PD management as a form of oral-systemic preventive strategy. Although many pharmacological glucose modulatory drugs exist in the market, in this article we offer a rationale for the implementation of metformin use in the clinics, as this is a cost-effective and globally available medication used as a first-line treatment for type 2 diabetes. Additionally, we further discuss the global health impact of dentists prescribing medication to treat oral disease and improve general health.

## Ageing in the periodontium

The aging process within the periodontium, encompassing the gingiva, cementum, periodontal ligament, and alveolar bone, involves several interconnected biological phenomena. Collagen changes, primarily associated with alterations in the composition, density, and structure of collagen fibres, emerge as a common theme affecting both the gingiva and the periodontal ligament (11, 12). This crucial structural component undergoes modifications with age, impacting the overall health and resilience of these tissues. Diminished vascularity and blood supply constitute another shared factor, influencing the gingiva and periodontal ligament. This reduction in blood flow can compromise oxygen and nutrient delivery, affecting tissue health and repair (13, 14). Additionally, both tissues are subject to the influence of chronic inflammation and immune changes associated with aging. This inflammaging phenomenon dysregulates the immune response and can contribute to increased susceptibility to periodontal diseases and impaired tissue healing in both the gingiva and periodontal ligament (15). These shared factors impact the structural support and regenerative potential of these tissues, underscoring the interconnected nature of the aging process within the periodontium.

Nevertheless, tissue-specific factors also contribute to the aging of individual components of this structure. For cementum, mineralization changes and reduced cellular activity emerge as key factors affecting its hardness and resilience. Moreover, accumulation of microdamage, influenced by mechanical stresses and normal masticatory forces, further compromises the structural integrity of the cementum and periodontal ligament (16, 17). Finally, ageing influences the alveolar bone due to reduced bone turnover [20], changes in mineralization (18), altered cellular activity (19), hormonal shifts (20), and responsiveness to mechanical loading (21), all of which lead to a complex interplay of factors affecting bone density and quality. Most specifically, and in need of further investigation, hormonal fluctuations, especially in postmenopausal women, have been linked to changes in periodontal tissues that are more favourable to periodontal disease breakdown (22).

## Plaque and glycemia during the lifespan: secrete double agents?

The presence of the oral microbiome is natural in every human being; therefore, the formation of plaque-biofilm is a natural biological process. Humans without oral hygiene intervention naturally accumulate plaque-biofilm around teeth, leading to PD (23); therefore, plaque levels need to be decreased to below the threshold of natural accumulation to prevent PD disease and development.

Similarly, blood sugar levels naturally fluctuate all day long, spiking when food is consumed. Continuous glucose build-up in the bloodstream can, over time, cause damage to human health even in normoglycemic humans; therefore, maintaining blood glucose at stable levels improves metabolic health preventing issues such as insulin resistance, obesity, and Type 2 diabetes (24); all of which have a direct impact on PD progression (4).

The catalyst for transitioning from stable chronic gingivitis to destructive periodontitis is still uncertain, yet it is understood that PD initiates due to biofilm accumulation, and inflammation is the mediator of the disease progression (25). Given the constant bacterial challenge from natural biofilm accumulation, the periodontal cells are subject to persistent oxidative stress and DNA damage (26, 27). Energy is necessary for constant DNA repair, precise cell turnover, and inflammatory regulation of the periodontal tissues. Glycolysis is the cellular metabolic pathway responsible for the breakdown of glucose to generate energy. Recent research suggests that glycolysis intermediates play a role in supporting DNA repair processes (28), however, little is known about this in the periodontal tissues. Yet, it is possible to infer that cellular glycolysis is important for periodontal stability as the impairment of a fine-tuned glycolytic cascade, due to insulin resistance or lack thereof, leads to increased risk of periodontitis breakdown (29). Further, continuous fluctuation of high peaks of blood glucose levels throughout the lifespan has been found to influence the development of conditions that share inflammatory risk factors with PD (30).

Although the biological interaction between glucose metabolism and plaque is not yet fully connected and elucidated, further investigations of these two natural daily fluctuant biological processes may unveil key information about the pathogenesis of PD. Moreover, systemic glucose modulatory strategies could be the missing link to enhancing PD prevention and simultaneously mitigating against other NCDs over one's lifespan.

## Current state of metformin use

Metformin is a cost-effective, globally available, and renowned medication used as a first-line treatment for type 2 diabetes (31). It operates by enhancing insulin sensitivity in the body's cells, thereby facilitating improved glucose utilization. Its appeal arises from its proven ability to regulate blood glucose levels without causing undue hypoglycemia, a common concern with some other antidiabetic medications.

Epidemiological studies have drawn the attention of researchers to therapeutic benefits of metformin beyond glycemic control. Metformin has been associated with a range of cardioprotective effects, reduced incidence of cancer and mortality, and increased longevity (31, 32). Adjoining these processes is the ability of Metformin to suppress inflammation, modulate the gastrointestinal microbiota, enhance osteoblast differentiation, repress osteoclast activity, and regulate stem cell aging, all of which indicate potential to highly impact PD management.

Aside from a wide range of *in vitro* research investigating the effects of metformin on the periodontium, several clinical studies exist looking at local administration of metformin gel when treating periodontitis (non-surgically), including non-diabetic patients (Table 1). Despite these positive findings, there was still no recommendation for topical metformin gel use in the management of stages I-III periodontitis by the European Federation of Periodontology and American Academy of Periodontology guidelines (44), due to it being an off-label use of the medication without a published formulation of the gel.

**Table 1:** Clinical research—non surgical.

Adjunct to subgingival instrumentation												
Study characteristics		Population				Intervention, I		Comparison, C		Main outcomes		
Author/year	Design	Age range/sex	Smoking status	Diagnosis/definition	Details on NSPT	N pts/sites with outcomes	Mode of delivery	N pts/sites with outcomes	Mode of delivery	Measures	Follow-up time points	Significant intergroup findings, *p* < 0.05
Arslaan et al. (33)	P	30–50; MF both included, ratio not reported	Not reported	LChP, 7 mm > PD ≥3 mm, 5 mm > CAL ≥1 mm	Excluded PI >2 or mSBI >2 or those using mouthwash used prior to SRP No details provided on SRP	28	1% MET gel (MET added to gel containing poloxamer 407, laponite, sodium benzoate and 2% carboxylmethylcellulose) on day 0 and 45	28	1% alendronate gel (formulation not reported) on day 0 and 45	PD	Day 45, day 90	I < C *p* = 0.011 (day 45) I < C *p* < 0.001 (day 90)
										CAL	Day 45, day 90	I < C *p* < 0.001 (day 45) I < C *p* < 0.001 (day 90)
										TNFa in GCF (ELISA)	Day 90	I < C *p* = 0.036 (day 90)
Bashir et al. (34)	SM	25–55; MF equal but N pts not reported	Non-smokers	ChP, ≥3 sites of PD ≥5 mm+RAL ≥3 mm + Radiographic BL	Supra/subgingival scaling Single visit SRP until root surface smooth + clean. Gracey curettes/US No prescription antiplaque agents Advised not to brush dressing Advised modified Bass technique, soft bristled brush and fluoridated toothpaste BD	16 sites	Normal saline irrigation + 1% MET gel (MET added to gellan/mannitol gel containing glycerine, sucralose, citric acid, methylparaben and sodium citrate) + periodontal dressing for 7 days	16 sites	C1: Normal saline irrigation + periodontal dressing for 7 days	PD	1 month, 3 months, 6 months	I ⇔ C1 ⇔ C2 *p* < 0.001 (3 months) I ⇔ C1 ⇔ C2 *p* < 0.001 (6 months)
								16 sites	C2: Normal saline irrigation + 1.2% simvastatin gel (simvastatin added to gel containing methylcellulose) + periodontal dressing for 7 days	RAL	1 month, 3 months, 6 months	I ⇔ C1 ⇔ C2 *p* < 0.001 (3 months) I ⇔ C1 ⇔ C2 *p* < 0.001 (6 months)
Shah et al. (35)	SM	20–60; 15M5F	Non-smokers	ChP, PD 5–7 mm present bilaterally, No vertical defects	SRP one week before local drug delivery. No details on SRP provided Advised not to brush near the area or use interdental aids for 1 week after local drug delivery	20 (20 sites)	1% MET gel (formulation not reported) + periodontal dressing (Coe-pacTM) for 1 week	20 (20 sites)	0.2% CHX gel (Cevitec) + periodontal dressing (Coe-pacTM) for 1 week	PD	1 month, 3 months	I < C *p* < 0.05 (1 month) I < C *p* < 0.05 (3 months)
										RCAL	1 month, 3 months	I < C *p* < 0.05 (1 month) I < C *p* < 0.05 (3 months)
Mirza et al. (36)	P	30–50; 31M26	Not reported	Moderate LChP, 7 mm > PD ≥3 mm, 5 mm > CAL ≥1 mm	Excluded PI >2 or mSBI >2 or those who used mouthwashes during the study No details provided on SRP	28	1% MET gel (MET added to gel containing poloxamer 407, laponite and sodium benzoate)	28	200mg doxycycline capsule STAT on day 0 and 100mg OD for 14 days	PD	Day 45, day 90	I < C *p* = 0.011 (day 45) I < C *p* < 0.001 (day 90)
										CAL	Day 45, day 90	I < C *p* < 0.001 (day 45) I < C *p* < 0.001 (day 90)
										TNFa in GCF (ELISA)	Day 90	I < C *p* = 0.009 (day 90)
Pundir et al. (37)	SM	>25	Non-smokers	ChP, PD ≥5 mm	SRP with Hand/US Advised no brushing/interdental aids in treated areas 1 week Supragingival scaling at recalls	40 (40 sites)	1% MET gel (MET added to gellan/mannitol gel containing citric acid, sucralose, propylparaben and methylparaben)	40 (40 sites)	Placebo gel (formulation not reported)	PD	3 months, 6 months, 9 months	I < C *p* = 0.002 (3 months) I < C *p* = 0.001 (6 months)
										CAL	3 months, 6 months, 9 months	I < C *p* = 0.001 (3 months) I < C *p* = 0.001 (6 months) I < C *p* = 0.047 (9 months)
Maatouq et al. (38)	P	30–60; 11M24F	Non-smokers	ChP, PD >4 mm	Phase I therapy (not specified) No details provided on SRP OH maintenance measures (not specified) once weekly for 6 weeks following SRP Soft plaque removed with cotton pledget at final visit before GCF collection	10	1% MET gel (MET added to gel containing carboxymethyl cellulose polymer, 0.18% methylparaben and propylene glycol) applied once weekly for 6 weeks	5	C1: Healthy gingivae, no treatment including no SRP	PD	6 weeks	C1 < C2 *p* < 0.001 (6 weeks) C3 < C2 *p* < 0.001 (6 weeks) I > C1 *p* < 0.001 (6 weeks) I < C2 *p* = 0.013 (6 weeks) I > C3 *p* = 0.015 (6 weeks)
								10	C2: No placebo, SRP only but repeated once weekly for 6 weeks	CAL	6 weeks	C1 < C2 *p* < 0.001 (6 weeks) C1 < C3 *p* < 0.001 (6 weeks) C3 < C2 *p* = 0.004 (6 weeks) I > C1 *p* < 0.001 (6 weeks) I > C3 *p* = 0.004 (6 weeks)
								10	C3: 2% clindamycin gel (clindamycin added to gel containing carboxymethyl cellulose polymer, 0.18% methylparaben and propylene glycol)	ALP in GCF (DGKC standard enzymatic colorimetric method)	6 weeks	C1 < C2 *p* = 0.005 (6 weeks) C3 < C2 *p* = 0.004 (6 weeks) I > C1 *p* = 0.029 (6 weeks) I > C3 *p* = 0.036 (6 weeks)
Mushtaq et al. (39)	P	25–55	Non-smokers	ChP, PD ≥5 mm, CAL ≥4 mm	Complete phase I therapy performed (not specified) Advised no brushing/interdental aids near treated areas for 1 week	15 (multiple mandibular/maxillary sites per patient)	1% MET gel (MET added to gellan/mannitol gel containing citric acid, sucralose, methylparaben, propylparaben, sodium citrate and raspberry flavour)	15 (multiple mandibular/maxillary sites per patient)	No placebo, SRP only	PD reduction	1 month reported	I > C *p* = 0.001 (baseline-1 month)
										CAL gain	1 month reported	I > C *p* = 0.001 (baseline-1 month)
Hasan et al. (40)	P	>20	Not reported	Periodontal diseases (not specified)	SRP one quadrant per week for 4 consecutive weeks	10	I1: 1% MET gel (MET added to 2% hydroxyethyl cellulose gel containing methyl paraben sodium, propylparaben sodium, EDTA and triethanolamine) applied 48hrs after each SRP visit	10	C1: No treatment, including no SRP	PD	Day 30	I1 < C1 *p* ≤ 0.001 (day 30) I1 < C2 *p* ≤ 0.001 (day 30) I2 < C1 *p* ≤ 0.001 (day 30) I2 < C2 *p* ≤ 0.001 (day 30)
										AL	Day 30	I1 < C1 *p* ≤ 0.001 (day 30) I1 < C2 *p* ≤ 0.001 (day 30) I2 < C1 *p* ≤ 0.001 (day 30) I2 < C2 *p* ≤ 0.001 (day 30)
						10	I2: 1% MET mouthwash (MET in mouthwash containing triethanolamine, glycerin, food dye and sodium benzoate) used at the end of each SRP visit	10	C2: No placebo, SRP only	TNFa in GCF (ELISA)	Day 30	C2 < C1 *p* ≤ 0.001 (day 30) I1 < C1 *p* ≤ 0.001 (day 30) I1 < C2 *p* ≤ 0.001 (day 30) I1 < I2 *p* ≤ 0.001 (day 30) I2 < C1 *p* ≤ 0.001 (day 30) I2 < C2 *p* ≤ 0.001 (day 30)
										PGE2 in GCF (ELISA)	Day 30	I1 < C1 p ≤ 0.001 (day 30) I1 < C2 *p* ≤ 0.001 (day 30) I1 < I2 *p* ≤ 0.001 (day 30) I2 < C1 *p* ≤ 0.001 (day 30) I2 < C2 *p* ≤ 0.001 (day 30)
										NO in GCF(ELISA)	Day 30	I1 < C1 *p* ≤ 0.001 (day 30) I1 < C2 *p* ≤ 0.001 (day 30) I2 < C1 *p* ≤ 0.001 (day 30) I2 < C2 *p* ≤ 0.001 (day 30) I2 < I1 *p* ≤ 0.001 (day 30)
Kasseem et al. (41)	P	36–55; MF both included, ratio not reported	Not reported	Moderate-to-severe ChP, 2–3 interproximal sites/quadrant with PD ≥5 mm, ≥20 teeth present	No details provided on SRP	10 (10 interproximal sites)	0.6% MET multiple layer mucoadhesive film (MET added to film containing 6% sodium carboxymethylcellulose and 4% thiolated sodium alginate)	10 (10 interproximal sites)	No placebo, SRP only	PD	6 months	I < C *p* < 0.05
										CAL	6 months	I < C *p* = 0.05
Santo Grace and Sankari (42)	P	Not reported	Not reported	ChP, PD>3 mm in 20 sites, ≥20 teeth present	No details provided on SRP	8 (upper first molar or, if missing, upper 7 teeth only)	1% MET gel (MET added to gellan/mannitol gel containing glycerine, sucralose, citric acid, methylparaben and sodium citrate)	8 (upper first molar or, if missing, upper 7 teeth only)	Placebo gel (formulation not reported)	PD	1 month	NS
										CAL	1 month	NS
Sreedhar et al. (43)	SM	34–64; 9M6F	Non-smokers	ChP, One site PD ≥5 mm per ≥3 quadrants	No details provided on SRP Advised brushing/rinsing with plain water only Selected quadrants cleaned with wet guaze to remove supragingival plaque day 30	15 (1 quadrant)	I1: 1% MET gel (MET added to gel containing carbopol 934P, 0.01% benzalkonium chloride and triethanolamine) on day 1 and placebo gel on day 30	15 (1 quadrant)	Placebo gel on day 1 and day 30 (formulation not reported)	PD	3 months	I2 < C *p* = 0.003 (3 months)
						15 (1 quadrant)	I2: 1% MET gel (MET added to gel containing carbopol 934P, 0.01% benzalkonium chloride and triethanolamine) on day 1 and day 30			CAL	3 months	I2 < C *p* = 0.002 (3 months) I2 < I1 *p* = 0.025 (3 months)
										ICTP levels in GCF (ELISA)	3 months	I1 < C *p* = 0.000 (3 months) I2 < C *p* = 0.000 (3 months) I2 < I1 *p* = 0.000 (3 months)

## Repurposing systemic metformin as an oral-systemic preventive medicine

PD management is in need of a holistic approach to address its immediate symptoms and underlying systemic risk factors as a preventive measure, prior to their establishment. Little is known about the systemic use of metformin on the periodontium of normoglycemic humans. However, systemic metformin has been shown *in vivo* to improve bone levels in a periodontal treatment model (45), and in a clinical trial to improve the gingival crevicular fluid inflammatory surrogates, simultaneously preventing weight gain in class 1 obese pregnant women (46). These research pieces indicate that systemic metformin use has potential to impact both oral and systemic management of complex NCD in normoglycemic humans.

**Figure 1:**
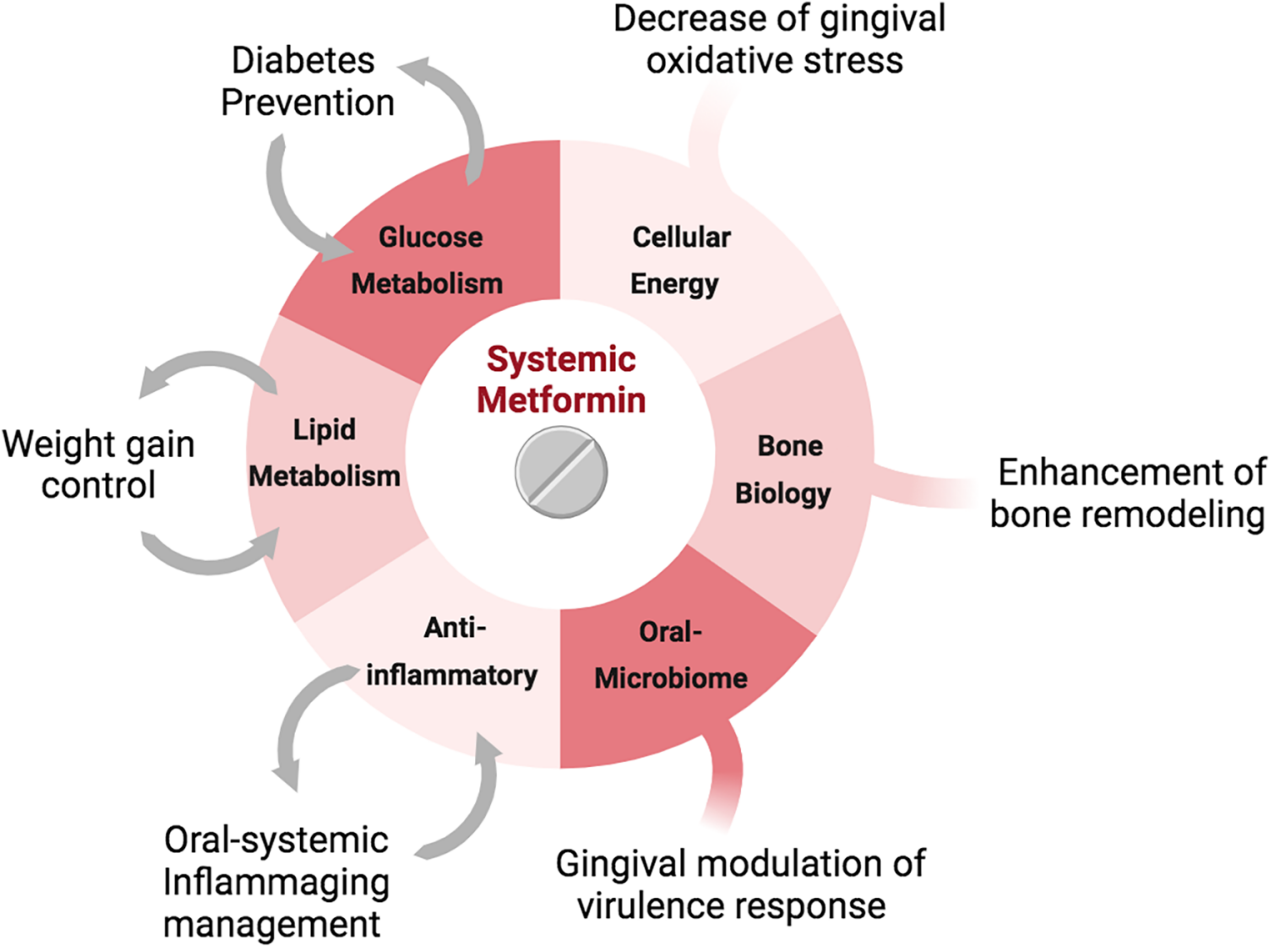
Metformin's mechanisms of action on oral and systemic health.

Further in a recent study, Neves et al. (47) investigated the efficacy of metformin in preventing and managing PD and its underlying associated risk factors, in healthy, non-obese, normoglycemic animals and humans (Figure 1). The *in vivo* results showed that systemic metformin use prior to inoculation of *Porphyromonas gingivalis* (*P. gingivalis*), halted *P. gingivalis*-associated periodontal bone loss, modulating the composition of the oral microbiome. Further, since metformin is considered a longevity drug, the study also investigated the role of metformin in preventing age-related periodontal bone loss, finding that long-term systemic metformin use reduced 54% of age-related periodontal bone loss. Using single-cell RNA sequencing analysis, Neves et al. (47) revealed that systemic use of metformin modulated the cellular energy metabolism related to glycolysis and mitochondrial function, as well as differentiation capacity of the gingiva. This resulted in enhanced preventive capacity against periodontal disease establishment, affecting the host-microbiome axis, even in the presence of dysbiosis.

Apart from the effect on the periodontium, the study also investigated how preventive systemic metformin impacted systemic associated risk factors, such as blood glucose levels and weight gain. The animals managed with metformin maintained significantly lower blood glucose levels compared to controls in all *in vivo* models. Furthermore, it demonstrated that PD induction increased systemic blood glucose levels, a feature of the disease that could potentially further explain the destructive synergy between PD and Diabetes. With regards to body weight, the *in vivo* results corroborate with the human weight gain prevention study, as animals on long-term systemic metformin were four times lighter than those on water.

Translationally, Neves et al. (47) ran the first randomized control trial using systemic metformin as an adjunct to periodontal disease treatment in normoglycemic and non-obese humans, as a first step to investigate the potential use of metformin in PD management. Using 850 mg of metformin once daily for 10 days, the study revealed comparable adverse effects to placebo and notable impacts on clinical periodontal parameters (overall additional PPD reduction of 0.3 mm and 1.09 mm in pockets ≥7 mm), similar to those of antibiotic therapies (48). Furthermore, systemic metformin treatment modulated systemic inflammation (IL-6 −0.9 pg/ml, hsCRP −2.15 mg/L), and systemic glucose metabolism, significantly enhancing insulin sensitivity compared to placebo. These findings suggest a potential for metformin to modulate co-morbidity risk factors and reduce age-related chronic conditions, laying a foundation for innovative approaches in PD management with metformin supplementation and this grant application.

The study also found that systemic metformin treatment modulated systemic inflammation and glucose metabolism, independently of any dietarian intake. Metformin administered as an adjunct to periodontal treatment stabilised fasting glucose and hsCRP, and improved insulin sensitivity, which together are markers associated with better metabolic health resulting in a lower likelihood of age-related chronic conditions.

## Medication or behaviour change?

Innovation in PD management demands a critical decision between implementing behavioural changes and medications, significantly impacting public health strategy. Patients striving to control their condition require a comprehensive approach, merging individual agency, preventive measures, and a holistic long-term well-being strategy.

Embracing lifestyle modifications, such as adopting a balanced diet, engaging in regular physical activity, and managing stress, can yield a myriad of benefits that extend beyond glucose control. Unlike medications, behaviour change empowers individuals to actively participate in their health journey, fostering long-term habits that promote overall well-being. Medications, on the other hand, offer a targeted and often swift approach to managing risk factors and mitigating the progression of various conditions. The benefit of medications in NCD prevention lies in their ability to address specific biochemical pathways or physiological processes, providing a reliable and measurable approach to managing risk factors.

Currently, oral hygiene (OH) stands as the sole strategy preventing PD establishment. However, its efficacy hinges on patient adherence, proficient OH techniques, and manual dexterity. OH proves limited for PD cases related to systemic diseases, genetic predisposition, or alterations in the oral environment due to detrimental habits.

Clinically, when considering adding to current PD prevention management strategies, dentists can emphasize the essential role of lifestyle changes in NCD prevention and PD impact. Moreover, our class can and should work in collaboration with Nutritionists despite the lack of consensus on the optimal PD management diet. However, dentistry's exclusive reliance on behavioural change, coupled with additional dietary-focused policies targeted at PD prevention to complement OH may overwhelm and discourage patients, especially those from less fortunate backgrounds focused on sustenance rather than dieting. It may also not have an additive effect to those who have no health services provided in their vicinity due to lack of health care workforce. Given dentistry's lack of a medication arsenal for PD's inflammatory biochemistry in secondary prevention, providing chemoprophylactic agents to control risk factors complements ongoing behavioural change efforts, preventing PD onset or progression and targeting NCD risk factors, ultimately impacting lifespan positively. This integrated approach addresses multifaceted challenges and access disparities, fortifying comprehensive PD management.

## Where is the line between dentistry and medicine?

An important consideration when strategizing systemic medication for oral diseases is whether dentistry is crossing the ethical boundary of its scope of practice. The boundary between medicine and dentistry lies in the direction of activity and the object of treatment, diagnosis and prevention. Medicine focuses on diseases of the entire human body, while dentistry focuses on the oral cavity and face, which have unique symptoms and management strategies. However, when an oral disease treatment has the scope to prevent systemic diseases, this boundary becomes blurry. While specific medical ethics may not directly apply to dental practice, fundamental values such as ethics, loyalty, and fiduciary relations should align across both professions (49). Here we try to shed light on the discussion about the autonomy within dentistry for prescription of systemic medication.

Medical practitioners can find signs of oral diseases but are not responsible for treating dental problems and so, should not attempt to manage a condition requiring dental skills unless they have appropriate training (50). Similarly, dentists can find signs of more severe disease when treating the oral cavity but also should not be responsible for treating the condition, requiring referring the patient to an appropriate medical practitioner. However, PD is highly common (more common than cardiovascular diseases), detrimental to wellbeing, affects quality of life and is directly linked to systemic health, including some life-threatening diseases (51). Therefore, when considering the impact of systemic interventions to prevent and manage PD and indirectly preventing systemic diseases, it could be argued that not intervening is a sign of neglect to overall health.

Crossing the boundary of oral-systemic health intervention is not a novelty in dentistry, dentists already prescribe systemic medications for various conditions related to oral health or systemic health concerns with oral manifestations. Yet, systemic medication prescription also comes with risk of misconduct, an example of this, is the misuse of antibiotics by dentists (52). Therefore, for systemic interventions to be rolled out for clinical use, tight collaboration with practitioners of medicine is essential for a safe practice. However, the current lack of autonomy to prescribe alternative medications for management of PD disease could hinder the progress and effectiveness of oral-systemic health intervention. With the necessary safeguards and collaboration with medical professionals, autonomy in prescribing medications to enhance oral health interventions would empower dentists to contribute significantly to the prevention and management of systemic diseases at the global level.

## Future directions for metformin repurposing

The integration of systemic metformin prescription for management and treatment of PD still needs further investigations and discussion for a concentrated widespread repurposing. Larger trials need to be done and investigations of the dose response for PD management need to be ascertained, as metformin prescription dosage is currently based on stabilising dysregulated systemic glucose metabolisms, not multi-morbidity prevention in normoglycemic patients. Further optimisation of the length of prescription is still to be ascertained so that the treatment can positively impact oral-systemic disease development and progression. Given that signs of multimorbidity prevention were seen when using metformin for PD management, the surrogate markers of these conditions, such as systemic glucose levels, lipid levels, and inflammatory trends on normoglycemic patients with PD need to be further investigated. Finally, investigations on patients with geriatric conditions associated with physical impairment affecting oral hygiene activities are needed as they could benefit from the indirect impact of metformin use for PD management. Together, these steps could lead to integration of PD disease management with metformin as a means to manage age-related morbidities prior to their development over a lifespan, impacting the global incidence of PD-associated risk factors, such as cardiovascular disease, obesity, diabetes, and cognitive decline.

## Conclusion

Here, we contextualize PD within the broader framework of non-communicable diseases (NCDs), highlighting its significant impact on systemic health and its association with ageing, underscoring the interconnectedness of PD with systemic health conditions and ageing-related changes in oral tissues and the microbiome. Further, we offered a comprehensive exploration of PD management and prevention strategies, emphasizing the potential of systemic glucose metabolism modulation, particularly through the use of metformin, as a promising avenue for oral-systemic preventive medicine. Finally, we addressed pertinent ethical considerations regarding the role of dentistry in systemic medication prescription, advocating for a collaborative approach between dental and medical professionals to ensure safe and effective oral-systemic health interventions, with implications for improving global public health outcomes.

## References

[1] Peres MA Macpherson LMD Weyant RJ Daly B Venturelli R Mathur MR Oral diseases: a global public health challenge. Lancet. (2019) 394(10194):249–60. 10.1016/S0140-6736(19)31146-8 31327369

[2] Savulescu J. Genetic interventions and the ethics of enhancement of human beings. In: Steinbock B, editor. The Oxford Handbooks of Bioethics. Oxford: Oxford University Press (2009). p. 516–35.

[3] Neves V Pugh J Savulescu J. Beyond oral hygiene, are capacity-altering, biologically based interventions within the moral domain of dentistry? Br Dent J. (2021) 231:277–80. 10.1038/s41415-021-3335-y 34508196

[4] Ramseier CA Woelber JP Kitzmann J Detzen L Carra MC Bouchard P. Impact of risk factor control interventions for smoking cessation and promotion of healthy lifestyles in patients with periodontitis: a systematic review. J Clin Periodontol. (2020) 47(Suppl 22):90–106. 10.1111/jcpe.13240 31912512

[5] Mathers CD Loncar D. Projections of global mortality and burden of disease from 2002 to 2030. PLoS Med. (2006) 3(11):e442. 10.1371/journal.pmed.0030442 17132052 PMC1664601

[6] Pihlstrom BL Michalowicz BS Johnson NW. Periodontal diseases. Lancet. (2005) 366(9499):1809–20. 10.1016/S0140-6736(05)67728-8 16298220

[7] Eke PI Dye BA Wei L Thornton-Evans GO Genco RJ. Prevalence of periodontitis in adults in the United States: 2009 and 2010. J Dent Res. (2012) 91(10):914–20. 10.1177/0022034512457373 22935673

[8] Hammel MC Stein R Kratzsch J Vogel M Eckert AJ Triatin RD Fasting indices of glucose-insulin-metabolism across life span and prediction of glycemic deterioration in children with obesity from new diagnostic cut-offs. Lancet Reg Health Eur. (2023) 30:100652. 10.1016/j.lanepe.2023.100652 37465325 PMC10350850

[9] Ishii M. What is good for the heart is good for brain glucose metabolism. Lancet Healthy Longev. (2023) 4(9):e448–9. 10.1016/S2666-7568(23)00167-8 37659422

[10] Longo VD Antebi A Bartke A Barzilai N Brown-Borg HM Caruso C Interventions to slow aging in humans: are we ready? Aging Cell. (2015) 14(4):497–510. 10.1111/acel.12338 25902704 PMC4531065

[11] Socransky SS Haffajee AD. Dental biofilms: difficult therapeutic targets. Periodontol 2000. (2002) 28(1):12–55. 10.1034/j.1600-0757.2002.280102.x 12013340

[12] Bosshardt DD Lang NP. The junctional epithelium: from health to disease. J Dent Res. (2005) 84(1):9–20. 10.1177/154405910508400102 15615869

[13] Nanci A Bosshardt DD. Structure of periodontal tissues in health and disease. Periodontol 2000. (2006) 40(1):11–28. 10.1111/j.1600-0757.2005.00141.x 16398683

[14] Jin K. A microcirculatory theory of aging. Aging Dis. (2019) 10(3):676–83. 10.14336/AD.2019.0315 31165010 PMC6538209

[15] Franceschi C Campisi J. Chronic inflammation (inflammaging) and its potential contribution to age-associated diseases. J Gerontol A Biol Sci Med Sci. (2014) 69 (Suppl 1):S4–9. 10.1093/gerona/glu057 24833586

[16] Lim WH Liu B Mah SJ Chen S Helms JA. The molecular and cellular effects of ageing on the periodontal ligament. J Clin Periodontol. (2014) 41(10):935–42. 10.1111/jcpe.12277 24888546

[17] Menicanin D Hynes K Han J Gronthos S Bartold PM. Cementum and periodontal ligament regeneration. In: Bertassoni L, Coelho P, editors. Engineering Mineralized and Load Bearing Tissues. Advances in Experimental Medicine and Biology. Vol. 881. Cham: Springer (2015). 10.1007/978-3-319-22345-2_12 26545752

[18] Ono R Katsumata A Fujikawa Y Takahira E Yamamoto T Kanamura N. Sex differences and age-related changes in the mandibular alveolar bone mineral density using a computer-aided measurement system for intraoral radiography. Sci Rep. (2024). 14:7386. 10.1038/s41598-024-57805-5 PMC10979020 38548856

[19] Marie PJ. Cellular regulation of bone remodeling. Calcif Tissue Int. (1985) 37(4):401–5. 10.1007/BF02553710 3930038

[20] Khosla S. Update on estrogens and the skeleton. J Clin Endocrinol Metab. (2010) 95(8):3569–77. 10.1210/jc.2010-0856 20685883 PMC2913030

[21] Turner CH. Three rules for bone adaptation to mechanical stimuli. Bone. (1998) 23(5):399–407. 10.1016/S8756-3282(98)00118-5 9823445

[22] Lee DJ Wu L Shimono M Piao Z Green DW Lee JM Differential mechanism of periodontitis progression in postmenopause. Front Physiol. (2018) 9:1098. 10.3389/fphys.2018.01098 30246792 PMC6113945

[23] Löe H Anerud A Boysen H Smith M. The natural history of periodontal disease in man. The rate of periodontal destruction before 40 years of age. J Periodontol. (1978) 49(12):607–20. 10.1902/jop.1978.49.12.607 282430

[24] Blonde L Umpierrez GE Reddy SS McGill JB Berga SL Bush M American association of clinical endocrinology clinical practice guideline: developing a diabetes mellitus comprehensive care plan-2022 update. Endocr Pract. (2022) 28(10):923–1049. 10.1016/j.eprac.2022.08.002 35963508 PMC10200071

[25] Curtis MA Diaz PI Van Dyke TE. The role of the microbiota in periodontal disease. Periodontol 2000. (2020) 83(1):14–25. 10.1111/prd.12296 32385883

[26] Zamora-Perez AL Zúñiga-González GM Gómez-Meda BC Lazalde-Ramos BP Ortiz-García YM Morales-Velazquez G Periodontal disease and nuclear and oxidative DNA damage. Insights into various aspects of oral health. InTech. (2017). 10.5772/intechopen.68446

[27] Kumar J Teoh SL Das S Mahakknaukrauh P. Oxidative stress in oral diseases: understanding its relation with other systemic diseases. Front Physiol. (2017) 8:693. 10.3389/fphys.2017.00693 28959211 PMC5603668

[28] Bhatt AN Chauhan A Khanna S Rai Y Singh S Soni R Transient elevation of glycolysis confers radio-resistance by facilitating DNA repair in cells. BMC Cancer. (2015) 15:335. 10.1186/s12885-015-1368-9 25925410 PMC4425929

[29] Chapple IL Genco R. Diabetes and periodontal diseases: consensus report of the joint EFP/AAP workshop on periodontitis and systemic diseases. J Periodontol. (2013) 84(4 Suppl):S106–12. 10.1902/jop.2013.1340011 23631572

[30] Flynn MC Kraakman MJ Tikellis C Lee MKS Hanssen NMJ Kammoun HL Transient intermittent hyperglycemia accelerates atherosclerosis by promoting myelopoiesis. Circ Res. (2020) 127(7):877–92. 10.1161/CIRCRESAHA.120.316653 32564710 PMC7486277

[31] Foretz M Guigas B Bertrand L Pollak M Viollet B. Metformin: from mechanisms of action to therapies. Cell Metab. (2014) 20(6):953–66. 10.1016/j.cmet.2014.09.018 25456737

[32] Zheng J Xu M Yang Q Hu C Walker V Lu J Efficacy of metformin targets on cardiometabolic health in the general population and non-diabetic individuals: a Mendelian randomization study. EBioMedicine. (2023) 96:104803. 10.1016/j.ebiom.2023.104803 37734206 PMC10514430

[33] Arslaan M Karim N Fatima M. Clinical efficacy of 1% metformin and alendronate intra-pocket gel in moderate localized chronic periodontitis. Rawal Med J. (2022) 47(1):112–5.

[34] Bashir S Gopalakrishnan D Martande S. Locally delivered 1.2% simvastatin gel and 1% metformin gel in chronic periodontitis patients. Saudi J Oral Dent Res. (2022) 7(8):182–91. 10.36348/sjodr.2022.v07i08.001

[35] Shah K Parikh H Duseja S. Comparative evaluation of metformin gel and chlorhexidine gel as adjunct to scaling and root planning in the treatment of chronic periodontitis: a clinical study. Int J Dentistry Res. (2022) 7(3):63–7. 10.31254/dentistry.2022.7305

[36] Mirza M Karim N Baksh Khadri W Asghar S. Clinical efficacy of 1% metformin gel and systemic doxycycline in chronic periodontitis: a randomised clinical trial. J Liaquat Uni Med Health Sci. (2021) 20(3):204–8. 10.22442/jlumhs.2021.00844

[37] Pundir AJ Gupta S Pundir S Bhatnagar S Agrawal N Sultana N. Evaluation of efficacy of subgingival delivered 1% metformin gel as an adjunct to scaling and root planing in the treatment of chronic periodontitis. Int J Med Sci Curr Res. (2021) 4(1):225–33.

[38] Maatouq AG Elshinnawi UM Raafat IM Mahsoub N. Therapeutic efficacy of locally delivered metformin gel versus clindamycin gel in chronic periodontal diseases. Mansoura J Dentistry. (2019) 6(24):10–4. 10.21608/mjd.2019.199352

[39] Mushtaq I Shukla P Malhotra G Dahiya V Kataria P Joshi CS. Comparative evaluation of 1% metformin gel as an adjunct to scaling and root planing in the treatment of chronic periodontitis with scaling and root planing alone: a randomised controlled clinical trial. Int J Oral Care Res. (2018) 6(2):79–88.

[40] Hasan F Najam R Simjee SU. Metformin- a non-conventional drug for the local treatment of periodontal diseases: a randomized clinical control study. Med Forum. (2017) 28(6):162–6.

[41] Kasseem AA Elsayed Issa DA Kotry GS Faird RM. Thiolated alginate-based multiple layer mucoadhesive films of metformin for intra-pocket local delivery: in vitro characterization and clinical assessment. Drug Dev Ind Pharm. (2017) 43(1):120–31. 10.1080/03639045.2016.1224895 27589817

[42] Santo Grace U Sankari M. Effect of locally administered 1% metformin gel in the treatment of chronic periodontitis. J Pharm Sci Res. (2017) 9(9):1463–5.

[43] Sreedhar A Sikkander S Pai J Walvekar A Baramappa R Varkey A. Clinical and biochemical evaluation of efficacy of 1% metformin gel as an adjunct to SRP in chronic periodontitis- a split mouth placebo controlled study. Int J Sci Res. (2017) 6(12):78–82.

[44] Sanz M Herrera D Kebschull M Chapple I Jepsen S Beglundh T Treatment of stage I-III periodontitis-the EFP S3 level clinical practice guideline. J Clin Periodontol. (2020) 47 Suppl 22(Suppl 22):4–60. 10.1111/jcpe.13290. Erratum in: J Clin Periodontol. 2021 Jan;48(1):163. 32383274 PMC7891343

[45] Araújo AA Pereira ASBF Medeiros CACX Brito GAC Leitão RFC Araújo LS Effects of metformin on inflammation, oxidative stress, and bone loss in a rat model of periodontitis. PLoS One. (2017) 12(8):e0183506. 10.1371/journal.pone.0183506 28847008 PMC5573680

[46] Zúñiga Curz CA Calzada Mendoza CC Miranda Mondragón ID Bustamante Bacame A Portilla Robertson J Ocharán Hernández E. Efecto del manejo de la obesidad clase I con metformina sobre actividad de metaloproteinasas en pacientes con periodontitis crónica [effect of the management of class I obesity with metformin on metalloproteinase activity in patients with chronic periodontitis]. Nutr Hosp. (2019) 36(5):1095–100. Spanish. 10.20960/nh.02602 31516010

[47] Neves VCM Satie Okajima L Elbahtety E Joseph S Daly J Menon A Repurposing metformin for periodontal disease management as a form of oral-systemic preventive medicine. J Transl Med. (2023) 21(1):655. 10.1186/s12967-023-04456-1 37814261 PMC10563330

[48] Teughels W Feres M Oud V Martín C Matesanz P Herrera D. Adjunctive effect of systemic antimicrobials in periodontitis therapy: a systematic review and meta-analysis. J Clin Periodontol. (2020) 47(Suppl 22):257–81. 10.1111/jcpe.13264 31994207

[49] Freukel DA Lurie Y. Responsibility and liability in health care: some differences between dentistry and medicine. Med Law. (2002) 21(3):605–15. PMID: . 12437206

[50] British Medical Association. Patients Presenting With Dental Problems. The British Medical Association (2019). Available online at: https://www.bma.org.uk/advice-and-support/gp-practices/gp-service-provision/patients-presenting-with-dental-problems (Accessed November 01, 2023).

[51] The Economist. Time to Take Gum Disease Seriously: the Societal and Economic Impact of Periodontitis. Available online at: https://impact.economist.com/perspectives/healthcare/time-take-gum-disease-seriously-societal-and-economic-impact-periodontitis (Accessed November 01, 2024).

[52] Bajalan A Bui T Salvadori G Marques D Schumacher A Rösing CK Awareness regarding antimicrobial resistance and confidence to prescribe antibiotics in dentistry: a cross-continental student survey. Antimicrob Resist Infect Control. (2022) 11(1):158. 10.1186/s13756-022-01192-x 36503570 PMC9741920

